# Incorporating MCDA into HTA: challenges and potential solutions, with a focus on lower income settings

**DOI:** 10.1186/s12962-018-0125-8

**Published:** 2018-11-09

**Authors:** Kevin Marsh, Praveen Thokala, Sitaporn Youngkong, Kalipso Chalkidou

**Affiliations:** 1 Patient Centred Research, Evidera, London, UK; 2 ScHARR, University of Sheffield, Sheffield, UK; 3 Faculty of Pharmacy, Mahidol University, Bangkok, Thailand; 4 School of Public Health, Imperial College, London, UK; 5 Center for Global Development, London, UK

**Keywords:** Multicriteria decision analysis, Health technical assessment, Lower and middle-income countries

## Abstract

**Background:**

Multicriteria decision analysis (MCDA) has the potential to bring more structure and transparency to health technology assessment (HTA). The objective of this paper is to highlight key methodological and practical challenges facing the use of MCDA for HTA, with a particular focus on lower and middle-income countries (LMICs), and to highlight potential solutions to these challenges.

**Methodological challenges:**

Key lessons from existing applications of MCDA to HTA are summarized, including: that the socio-technical design of the MCDA reflect the local decision problem; the criteria set properties of additive models are understood and applied; and the alternative approaches for estimating opportunity cost, and the challenges with these approaches are understood.

**Practical challenges:**

Existing efforts to implement HTA in LMICs suggest a number of lessons that can help overcome the practical challenges facing the implementation of MCDA in LMICs, including: adapting inputs from other settings and from expert opinion; investing in technical capacity; embedding the MCDA in the decision-making process; and ensuring that the MCDA design reflects local cultural and social factors.

**Conclusion:**

MCDA has the potential to improve decision making in LMICs. For this potential to be achieved, it is important that the lessons from existing applications of MCDA are learned.

## Background

Health care decision making bodies across the world face the challenge of choosing which technologies to fund with scarce resources. This is supported by health technology assessment (HTA), to estimate the value for money of technologies. While factors other than cost and health benefit are acknowledged by HTA agencies [1], these are not always precisely measured, and their value is not formally assessed [2]. This has led to calls for methods capable of capturing the broader set of value of interest to payers, including consideration of, for instance, equity issues, burden of disease and a broader set of social benefits.

The past few years have witnessed a surge of interest in the use of multi-criteria decision analysis (MCDA) in HTA [3]. MCDA is a collection of approaches that support decision making by taking explicit account of multiple criteria. They guide decision makers through the process of agreeing what factors are relevant to a decision, measuring performance of options against these criteria, and understanding the trade-offs between values that may be conflicting. Without such structure, priority setting processes can be ad hoc, and not include all relevant stakeholders [2]. This is particularly the case in lower and middle income countries (LMICs), where priority setting tends to be more complex due to there being limited evidence to inform decisions, countries’ fragile institutional capacity and the dominant influence of policy makers’ opinions and international donor agencies [4, 5]. In these circumstances, MCDA can support the quality of decision making and increase transparency and consistency [6].

A growing number of decision making bodies and HTA agencies in high income countries (HICs) are either using or starting to explore these approaches to improve their transparency and accountability, including: Germany (Institute for Quality and Efficiency in Health Care [IQWiG]; [7] Italy (Lombardy); [8] South Korea; [9] Hungary; [10] UK (National Institute for Health and Care Excellence [NICE] highly specialised technology [11]). While a lot of attention is given to HIC’s use of MCDA, an unusually large proportion of published MCDAs for HTA are undertaken in LMICs. Two recent reviews of MCDAs in health care [3, 12] identified 10 studies of MCDAs used to inform HTA in LMICs, including: formulary management in Malaysia [13] and Cote d’Ivoire [14], and priority setting in Brazil [15], Thailand [16, 17], Ghana [18, 19], Nepal [20], Morocco and Tunisia [21] and South Africa [22]. In one of these reviews, nine out of a total of 23 published MCDAs for HTA were undertaken in LMICs [3].

The objective of this paper is to provide an overview of the methodological and practical challenges facing the use of MCDA for HTA, with a particular focus on LMICs, and to highlight potential solutions to these challenges. The next section outlines methodological challenges facing the application of MCDA for HTA. The following section then considers some of the practical challenges to implementing MCDA, specific to LMICs. Four case studies of the application of MCDA in LMICs are referred to throughout these sections.

### Methodological challenges

The use of MCDA for HTA is still in its infancy, and guidelines on good practice have only recently been published [24]. As guidelines are developed, it is becoming clear that many examples of MCDA for HTA, whether in LMICs or otherwise, are not applying good practice. It is important that those implementing MCDA in LMICs learn the lessons from this experience, and are aware of the current, more general methodological debates in the application of MCDA for HTA. This section identifies some of these challenges and points towards good practice that can help practitioners address them.

### Positioning on the socio-technical continuum

Perhaps the first challenge facing the design of an MCDA is to determine where on the socio-technical continuum the MCDA design should sit, ranging from purely deliberative to fully quantified/algorithmic [25]. The technical element of MCDA addresses the analytical questions: how to ensure criteria properties and criteria set properties comply with good practice, how to measure performance against these, how the criteria are weighted, and how performance and weights are aggregated. The social element of MCDA is concerned with which stakeholders are involved in the MCDA, and when and how they contribute. There is no a priori optimal position on this spectrum. Technical and social elements need to work in concert to achieve the aims of the MCDA, and the appropriate combination of elements will depend on the decision problem.

It has been argued that HTA is an ethical problem, and that MCDA can support HTA by facilitating the deliberation required for stakeholders with diverse perspectives to learn from one another and achieve agreement [26]. That is, the appropriate MCDA design for HTA emphasises fair process, argumentation, iteration and systematic thinking, alongside the technicalities of preference elicitation and aggregation methods. However, whether this is the case will depend on the decision problem facing HTA agencies. For instance, the MCDA required by IQWiG to support economic evaluation would probably be considered towards the more technical end of the spectrum, involving the use of a discrete choice experiment to elicit the preferences of large samples of patients [7].

The case studies highlighted in this paper illustrate a range of socio-technical approaches to MCDA for HTA. All involved significant stakeholder engagement in the design, implementation, and interpretation of the results of the MCDA. The stakeholders involved varied; for instance, criteria weights were elicited from policy makers in Thailand (Table 1), Indonesia (Table 2) and Ghana (Table 3), but from the general population in Colombia (Table 4). In Ghana, Indonesia and Thailand, the results of the MCDA were discussed and validated by decision makers before they used the MCDA to support their decision making. In Colombia, the MCDA had a more direct impact on decisions, with the technologies that performed best in the MCDA being included in benefits package until the point at which the available budget was spent.

**Table 1:** Case study—Thailand [16, 23]

Thailand is a frontrunner in the use of MCDA to prioritise health interventions. Since 2009, the prioritisation of non-pharmaceutical products available under universal health coverage (UHC) has involved the following steps: (1) nomination of topics/interventions for assessment by seven groups of stakeholders, comprising policy makers, health professionals, civil society, academics, industries, general population and patient groups; (2) scoring of options against the selection criteria by the research team; (3) selection of topics/interventions for assessment by consultation panels of stakeholders representing the Thai health insurance system, policy makers and academics; (4) technology assessment of interventions by the research team; and (5) discussion of the assessment results and decision making by the SCBP. Final approval is sought from the subcommittee on health financing
The MCDA is embedded in a decision making institution, being initiated by the National Health Security Office (NHSO), the institute managing UHC. For instance, in 2009 the MCDA assessed 17 possible services for inclusion in UHC. The research team presented the results of the assessment of nine of these interventions to the SCBP, who recommended that three of these be considered for adoption under UHC

**Table 2:** Case study—Indonesia [29]

An MCDA was undertaken to inform the 5-year HIV/AIDs strategic plan in West Java province, Indonesia. Criteria and weights were agreed upon by a consultation panel, comprising 23 representatives from different government departments, community organisations, programme managers and researchers. A larger group of stakeholders proposed 50 interventions, which were scored by researchers. The consultation panel reflected on the results of the MCDA, incorporated other ethical considerations to prioritise investments and considered implementation, including who should fund and implement the prioritised interventions
The methods and results of the MCDA were included in West Java’s 5-year strategic document for HIV/AIDS control, which was approved by the governor in 2014. However, this was only a guidance document, and the extent to which it determines resource allocation is uncertain

**Table 3:** Case study—Ghana [18]

An MCDA was undertaken to guide the national Ministry of Health in Ghana in priority setting, by ranking 26 interventions. Specifically, the MCDA quantified the trade-off between equity, efficiency, and other societal concerns in health. A focus group of seven policymakers identified the relevant criteria for priority setting, including: the severity of the disease, the number of potential beneficiaries, the cost-effectiveness of the intervention, whether the intervention reduced poverty, and whether the intervention targeted a vulnerable population. A total of 63 policymakers participated in a discrete choice survey, and regression analysis was used to infer from their choices the weights associated with criteria
The priority-setting process was strongly embedded in the organisation context of the Ministry of Health to ensure its integration into the third Five Year Program of Work. Anecdotal evidence showed that policymakers used the study findings as part of the development of their Five Year Programme of Work

**Table 4:** Case study—Colombia [38, 39]

With the cost of medications and devices seen as a threat to the sustainability of the funding of the health care system, between 2011 and 2013 the Instituto de Evaluación Tecnológica en Salud (IETS) implemented an MCDA to inform the inclusion of technologies in the health benefits package. The Ministry of Health undertook a systematic review to identify criteria, from which a shortlist was selected by relevant stakeholders. Technologies are scored against the criteria using 5-point Likert scales by stakeholders including Ministry of Health staff, citizens and physicians. Weights were obtained from a survey of 200 people from the Colombian general population
The MCDA informed the decision about additions to the health benefits package in 2013. Technologies that were candidates for inclusion but did not make it into the benefits package in 2011, as well as technologies that the judiciary had made available to individual patients, made up the list of 314 technologies considered. The Ministry of Health prioritised 105 technologies for evaluation based on disease burden and the number of requests via tutela (the judicial mechanisms to request technologies not included in the benefits package). Based on the MCDA benefit-score and the available budget, 70 technologies were included in the benefits package

It is recommended that those designing an MCDA are familiar with the decision problem, including the relevant stakeholders, and the alternative MCDA methods available to support this decision problem. Decision makers should be engaged in defining the decision and the appropriate MCDA solution. Alternative approaches should be presented to decision makers, with an assessment of their relative merits, so that decision makers can input into the process of methods design. Regardless of where on the continuum the appropriate MCDA design is located, it is important to follow good practice recommendations relating to both the technical and social elements of MCDA [24, 27].

### Quantifying the benefits of technologies

By far the most prevalent aggregation function adopted by MCDAs for HTA is the additive one. This has the advantage of being analytically simple. However, such simplicity requires that the criteria set have certain properties. In particular, they need to be non-overlapping (i.e., avoiding the double counting of the value generated by an alternative) and preferentially independent (i.e., the weight attached to one criterion should not depend on the performance on other criteria).

Unfortunately, many applications of the additive model to HTA violate these requirements. Preferential dependence—when the weight attached to one criterion is dependent on the performance on another—is a common problem in MCDAs for HTA. Perhaps the most prevalent example of this is the additive combination of health gain and severity of disease, when we might reasonably expect the value of health gain to be dependent on disease severity [28]. Three of the four case studies presented in this paper are subject to this potential type of preferential dependence, including both severity of disease and effectiveness [Thailand (Table 1), Colombia (Table 4)] or cost-effectiveness [Ghana (Table 3)]. In the presence of preferential dependence, the additive model is invalid and two options are available: adopt a different model structure that reflects the non-additive relationship between criteria, or update the criteria to ensure they are independent [24].

### Measuring opportunity cost

HTA requires not only that the benefits of technologies are estimated, but also that opportunity cost is estimated using the same measure. This is the case whether HTA is supported by MCDA or other methods, such as cost-effectiveness analysis. Where HTA involves a technology-by-technology evaluation, we can distinguish three approaches to measure willing to pay (WTP) for gains measured on the MCDA-benefit scale. First, stated preference methods can be used to elicit stakeholders WTP for the benefits of technologies. Many MCDAs for HTA include cost or budget impact as a criterion. While not explicitly acknowledged by these studies, this is a stated preference approach. The weight elicitation stage of the MCDA essentially elicits stakeholder WTP for benefits. Two of the case studies presented in this paper include cost-effectiveness as a criterion [Ghana (Table 3) and Indonesia (Table 2)].

This approach, however, poses significant challenges. Oftentimes these MCDAs elicit weights separate from scales of performance. For instance, asking stakeholders to estimate the relative importance of ‘budget impact’ and ‘health gain’. However, without more precise definitions of these criteria, including precise scale ranges, it is almost impossible to provide a precise WTP estimate. Even with more precise definitions, it is questionable whether stakeholders would have the necessary knowledge of the returns on current expenditure, or the cognitive capacities to translate this into reliable responses to preference elicitation exercises. For instance, the relative weight attached to cost and benefits need to reflect the willingness to pay for different benefits and how this changes in different circumstances. This challenge is exacerbated when the criterion is cost-effectiveness, as many different changes in effectiveness and cost could be reflected in a single cost-effectiveness ratio.

These concerns form the basis of the recommendation that MCDAs should not include cost or budget impact as a criterion, and instead that MCDA should be used to estimate the benefits of technologies. This is the approach adopted in the other two case studies [Thailand (Table 1) and Colombia (Table 4)]. This broader benefit measure could then be compared against the incremental cost of the technology to form a new incremental cost-effectiveness ratio (ICER). Then, akin to the current application of the cost-effectiveness analysis, the efficiency of a technology is assessed by comparing an ICER against an opportunity cost threshold. The broader benefit measure could be seen as replacing the quality-adjusted life-year (QALY)—though the QALY could still be one of the criteria making up this broader measure.

Two methods have been adopted to estimate such an opportunity cost threshold in the context of cost-effectiveness analysis [30]. Both could be applied in the context of MCDA, but doing so would pose challenges. First, an analysis of historic decisions to reveal decision makers’ preferences [31]. However, that no decisions would have been made using MCDA at the point of designing an HTA approach makes this unfeasible. Second, empirical estimates of the changes in benefits resulting from changes in expenditure [32]. This too faces practical challenges. Efforts to apply this approach to estimate the cost-effectiveness threshold have been criticised for the number of assumptions required to make up for the limitations in the available data [33]. More challenges would be faced applying this approach to MCDA.

Faced with similar challenges when implementing cost-effectiveness analysis, it has been proposed that LMICs adopt a strategy of extrapolating opportunity cost estimates from wealthier settings [34] or estimating opportunity cost based on local income levels [35], especially in the absence of WHO guidance on the matter. The same approach cannot, however, be applied in the context of MCDA until these wealthier settings identify estimates of their own MCDA-based opportunity cost.

Alternatively, those designing HTA systems might avoid a technology-by-technology assessment process. If HTA involves the assessment of all technologies simultaneously, the challenge of estimating opportunity cost is removed. In that instance, a decision rule could take the form of investing in technologies in order of their ICERs until the budget is expended (see for instance Airoldi et al. 2011 [36]). In this instance, MCDA can be applied as part of programme budgeting marginal analysis [37]. This is the approach adopted in three of the four case studies presented in this paper—Thailand (Table 1), Indonesia (Table 2), and Ghana (Table 3). However, in none of these instances was the whole health care budget or the whole benefits package considered, something which would be practically impossible. Instead, this application of MCDA is practically limited to individual care pathways or vertical programmes, such as HIV. As a consequence, opportunity costs issue remains which are not considered by the analysis.

## Practical challenges

Linked to the methodological challenges presented above, there are a number of practical challenges related to the application of MCDA as part of an HTA process. Many of these apply to HTA generally, whereas some are specific to MCDA. Most are also more acute in resource constrained settings, whether in low income settings or middle income economies with budding HTA mechanisms in the context of insurance agencies.

### Informational requirements

Sourcing inputs in systems with less reliable information systems, including limitations in context-specific information, is a major challenge with HTA. Regardless of whether MCDA is employed, HTA will require data on (unit) costs, resource use, epidemiology, comparative effectiveness specific to the setting, and outcomes data including patient-reported outcomes. Distribution of health and access to health care is another type of information required by HTA, and while not specific to LMICs, it is particularly important in LMICs.

The literature on the development of HTA systems in LMICs identifies potential solutions to gathering this data [40, 41]. Promising avenues for the collection of these data is collating and adapting evidence on effectiveness from other settings; and the use of expert opinion to help translate evidence into the local setting and fill gaps in the evidence [42–44]. In the Thai case study (Table 1) there was a lack of local scientific information on one of the topic selection criteria ‘targeting the poor and those with rare diseases’. Performance against this criterion was determined on the basis of the Thai experts’ opinion and international guidelines. Though caution is advised, as there are also examples of the application of external data to local settings that have not been successful, such as in the case of the Filipino health insurance scheme [45].

Faced with limited information, MCDA has a number of benefits. First, it is able to accommodate criteria defined and measured using expert opinion. Second, it allows for the formal consideration of the impact of uncertainty on the analysis. Relying on expert opinion, or generalising from evidence collected in other jurisdictions, introduces uncertainty into HTA. By breaking up HTA into its components parts—criteria, performance and valuation—MCDA enables the impact of uncertainty in any of these components on the results of a HTA to be assessed [46]. This will allow a formal assessment of whether this uncertainty impacts on the conclusion of the MCDA.

MCDA also involves information requirements of its own, such as the need to attach appropriate weights/scores reflecting social preferences. Indeed, weight elicitation and how transparent and understandable those are to decision makers and the broader public is a general practical challenge for MCDA. Some criteria used in MCDA, like equity, are difficult for non-experts to understand. The elicitation of weights is a specialist activity, prone to bias, pointing to the need for appropriate technical capacity—the topic of the next section.

### Technical capacity obstacles—different types of capacity shortfalls

Related to the informational/evidential challenges raised above, the relative lack of well-trained people who can initiate, manage and apply the whole process of HTA/MCDA is a practical obstacle to its routine introduction, especially in LMIC settings. Technical capacity required to deliver an MCDA includes: knowledge of good MCDA practice, such as the properties of criteria required to implement an additive model (see above); modelling skills, though most MCDA models are not technically demanding; and preference elicitation expertise. The latter may include techniques such as decision conferencing, which encourage stakeholders to express their views, facilitate the generation of consensus and help inform the model as it develops on-the-spot with continuous display of the model’s results through a highly ‘interactive and iterative group process’ [47].

The ability to commission and quality assure MCDAs from the right groups on behalf of decision makers, and of translating the findings into policy language and specific and actionable recommendations, is an important one which is often lacking in LMICs. This said, there are examples of countries that within a 5- to 10-year period have built a significant capacity, technical, clinical, political and administrative, in support of HTA including MCDA, such as Thailand with the Health Intervention and Technology Assessment Program (HITAP) [16, 23, 48, 49]. Also, regional initiatives such as HTAsiaLink can help countries work together, oftentimes pooling experiences as well as expertise, to support local decision making efforts [41, 50]. Finally, while training programmes at local universities are often in short supply, they are available. Mahidol’s HTA postgraduate programmes in Southeast Asia is one such example [51]. Its programmes were established in 2016, partly with funding from iDSI [52], to build up social and intellectual capital for, ultimately, strengthening and sustaining capacity for HTA research and implementation, and support UHC in LMICs.

### Issues of governance, institutions and consistency in decision making

The MCDA literature tends to emphasise the technical aspects of implementation, such as those describe above. Relatively little attention is paid to the extent to which the MCDA is embedded in the institutional policy-making process, something that is necessary to ensure that its results will actually be used [29]. Five of the nine examples of MCDAs for HTA in LMICs identified by Marsh et al. [3] were applied in an institutional setting; three being used to support decisions [16, 18, 22] and two being exploratory studies involving officials [14, 17].

The embedding of MCDA in relevant decision-making processes requires established or mature enough institutions. First, if such institutions do not exist, the ability to engage relevant stakeholders to elicit their input into the implementation of the MCDA, as well as to communicate the results of the MCDA, can be undermined by limited trust in public institutions. Second, institutions are required that are able to carry out or at least influence budgetary allocation (or de-allocation). Obstacles to implementing decisions include: weak regulation; unreliable payment mechanisms; or misaligned ones such as ‘Fee For Service’ which can encourage supply-induced demand, and a lack of effective performance-based contracts. Additional practical challenges include the fragmentation of decision making structures and of financing cash flows, including oftentimes between central and federal or public and private sectors, and between decision makers at the Ministry of Health and various insurance schemes as well as significant out of pocket spending.

However, where policy makers develop an interest in HTA, there are significant opportunities for progress in terms of institutionalising HTA-type approaches. This can be facilitated by creating positions within ministries and insurance funds for supporting institutions, and signalling to academic institutions, the health care products’ industry and international players that the government is determined to adopt a more explicit and evidence informed approach to priority setting. India, through the Indian Council of Medical Research and the Department for Health Research at the Union level, and China, under the leadership of the National Health and Family Planning Commission, both recently announced the launch of HTA institutions and networks, respectively, to support the transition to UHC [53–55]. Another encouraging experience can be found in Thailand. The Thai experience highlights the need for senior figures from powerful bodies to champion MCDA. The introduction of MCDA/HTA in Thailand was supported by the chair of the sub-committee of UHC benefit package development and the chair of the committee of National List of Essential Medicines, both of whom realised the importance of evidence-informed decision making [16, 48, 56].

The role of international donors in LMICs creates both challenges to and opportunities for the embedding of MCDA in decision making. To the extent to which donors use MCDA to support their own decision making, their demands for related evidence can drive the adoption of MCDA in LMICs. Further, donors or funding conduits such as the Global Fund [57], can contribute to the building of in-country capacity, technical and institutional, for commissioning, carrying out and applying MCDAs to decisions about countries’ own spending, which is increasingly important as countries transition away from aid. The potential for applying MCDA in the context of specific diseases and conditions, with its structured approach to stakeholder engagement and explicitness about consideration of issues such as equity and human rights, ought to make its application attractive to funding channels such as Gavi, which funds vaccines, and the Global Fund, which is responsible for funding interventions targeting HIV/AIDS, TB and malaria in developing countries.

On the other hand, donor’s influence may have a detrimental impact on the adoption of MCDA. Being explicit about opportunity costs or costs in general can often seem controversial in the global development world, acknowledging budgetary constraints is often deemed unethical and economists demonised [58, 59]. Further, when funding pots are limited to vaccines or a certain disease such as TB or HIV, issues of allocative efficiency become less relevant in the short term, at least for the foreign budget holders making investment choices in poorer countries. We hope however, that as the value for money rhetoric [60] finds its way into government policies in rich and poorer countries, MCDA will offer a means for considering in a systematic fashion most of the things that matter when allocation decisions are made, including costs and distribution.

### Cultural and social factors

MCDA presents the possibility of formally and transparently incorporating local cultural and social factors into decision making. It is not only the health and economic impacts of interventions that are of interest in HTA, but also the social, ethical, and institutional implications of a technology. MCDA can help HTA consider these factors, by incorporating them into the criteria list and weighting them to reflect their relative importance to stakeholders. For instance, the example of the use of MCDA to inform UHC in Thailand demonstrates the influence of social values throughout the MCDA, from the topic selection stage, through the inclusion of health technologies that are not equally accessible throughout the country or across different health facilities, to the criteria reflecting equity and social implications, with preference given to health problems inflicting the poor or minority groups with rare diseases [16]. Another example is the Indonesian case study (Table 2), which includes a criterion measuring the impact of interventions on the stigma associated with being HIV positive.

Furthermore, by structuring stakeholder input, and by explicitly incorporating cultural and social factors into decision criteria, MCDA reduces the risk that decision making is unduly influenced by local power structures. However, adopting MCDA does not guarantee stakeholders’ representation in decision making. In LMICS, the technical nature of MCDA may result in deliberations being dominated by highly educated people with the views of the majority less well reflected [61]. It is important that this is considered when designing the MCDA process, so that the appropriate support is provided to allow everyone to participate.

## Conclusion

MCDA is increasingly being considered as a means to address some of the limitations with existing HTA methods. In particular, MCDA offers a means to more formally and transparently capture the multiple factors relevant to HTA. Despite the attention given to debates in high income countries, an unusually large proportion of published MCDAs for HTA are undertaken in LMICs. This may be explained by the lack of established HTA methodologies in LMICs, allowing recent innovations to take hold. This literature, and broader debates about the application of MCDA in health care, point to a number of lessons for those implementing MCDA in LMICs:

1. It is important that new efforts to implement MCDA in LMICs don’t replicate the technical limitations of existing applications, such as ignoring the preferential dependence between criteria (e.g. severity of disease and health gain). Those implementing MCDAs should familiarize themselves with recent good practice guidance.
2. The estimation of opportunity cost for use in an MCDA-framework that undertakes technology-by-technology evaluations is a significant challenge. This can be somewhat addressed if the decision problem can be defined to be prioritizing which of many interventions to fund from a fixed budget.
3. A lack of evidence in LMICs can partly be addressed through generalizing from evidence generated in other settings and the use of expert opinion, in combination with an analysis of the impact of uncertainty on the conclusions of the MCDA.
4. Investment in technical capacity is necessary to ensure a rigorous implementation of the MCDA, in particular the design of the overall MCDA approach, and the elicitation of weights.
5. If it is to impact decision making, it is important that the design of the MCDA reflects the local decision problem, including being embedded in decision making institutions, and engaging relevant stakeholders in design, implementation, and interpretation.

## Abbreviations

CUA: cost-utility analysis
DSU: Decision Support Unit
HIC: high income country
HITAP: Health Intervention and Technology Assessment Program
HTA: health technology assessment
ICER: incremental cost-effectiveness ratio
IETS: Instituto de Evaluación Tecnológica en Salud
IQWiG: Institute for Quality and Efficiency in Health Care
LMIC: lower and middle income country
MCDA: multi-criteria decision analysis
NHSO: National Health Security Office’s
NICE: National Institute for Health and Care Excellence
QALY: quality-adjusted life-year
SCBP: sub-committee for the development of benefit package and service delivery
UHC: Universal Health Coverage
WTP: willingness to pay
